# Supplementary material for: Mesomelic dysplasias associated with the HOXD locus are caused by regulatory reallocations
Source: Nat Commun. 2021 Aug 18;12:5013. doi: 10.1038/s41467-021-25330-y (PMC8373931; doi:10.1038/s41467-021-25330-y)
Supplement: Supplementary file 1 — Supplementary Information [file 41467_2021_25330_MOESM1_ESM.pdf]

Supplementary Information for:

Mesomelic dysplasias associated with the *HOXD* locus are caused by regulatory reallocations

Christopher Chase Bolt, Lucille Lopez-Delisle, Bénédicte Mascrez,

and Denis Duboule

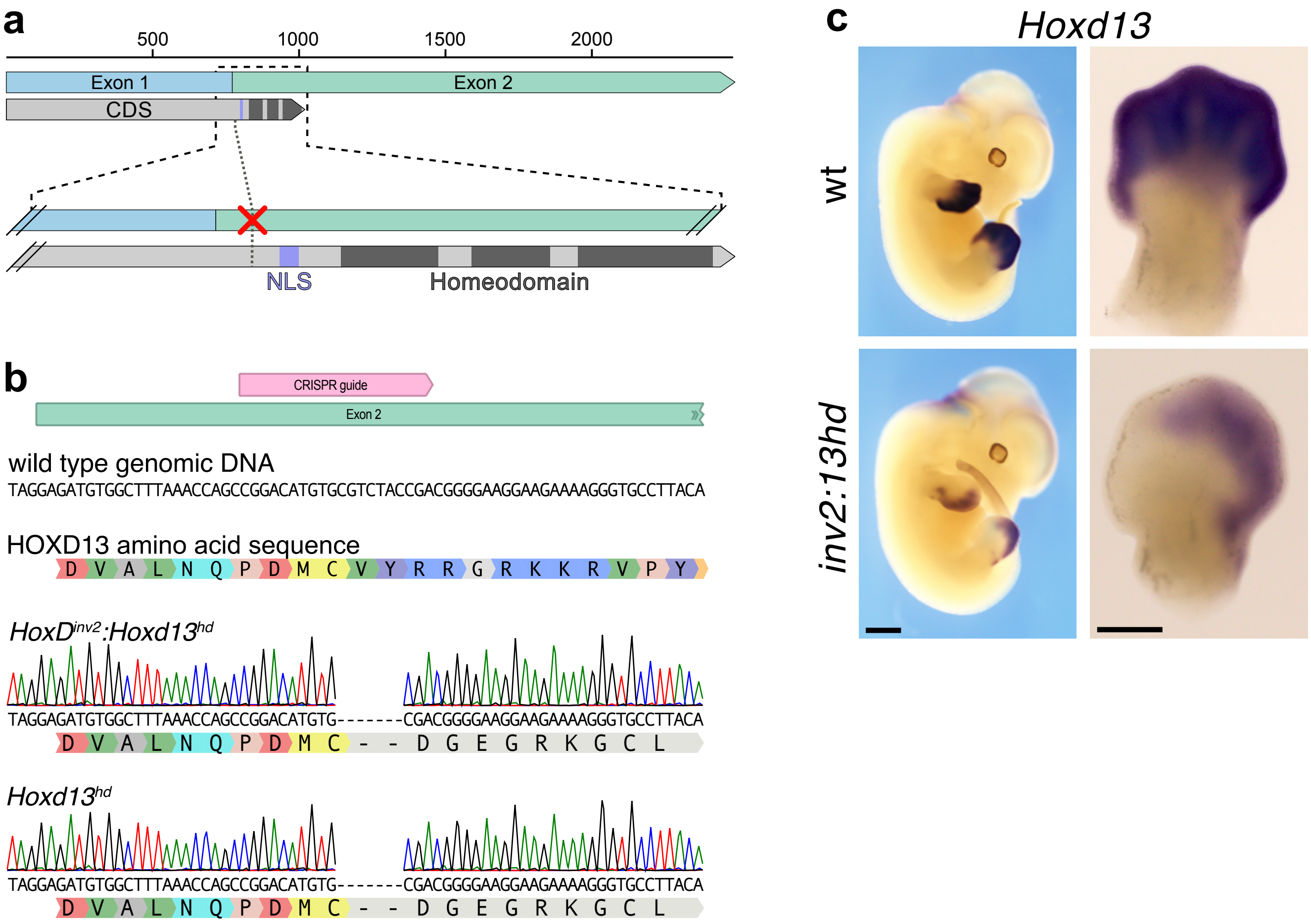

Bolt et al., 2021
Supplementary Figure 1

Supplementary Figure 1. Generation and validation of the *Hoxd13*^{hd} CRISPR-induced mutation. **a** Map of the *Hoxd13* spliced mRNA (top panel, blue and green) with the protein coding sequence below (grey), with the three alpha-helices of the homeodomain (dark grey). The bottom panel indicates the position of the CRISPR guide that is shown with a red X, N-terminal to the nuclear localization domain (NLS). **b** Two alleles were generated using the same CRISPR guide. The first one had a 7bp deletion in exon 2, in *cis* with the *HoxD*^{inv2} allele (*HoxD*^{inv2}*:Hoxd13*^{hd} referred to as *inv2:13hd*). The second allele had the same 7bp deletion, yet on the wild type chromosome (*Hoxd13*^{hd} referred to as *13hd*). In both cases, a frame-shift had occurred starting N-terminal to the NLS, which produced a non-functional homeodomain. **c** *In situ* hybridizations for *Hoxd13* on E12.5 wild type and *inv2:13hd* embryos, showing that the secondary mutation did not alter the location or the quantity of mRNA when compared to the initial *inv2* allele (see Figure 1c). Scale bar for whole embryos is 2mm and for limb is 0.5mm.

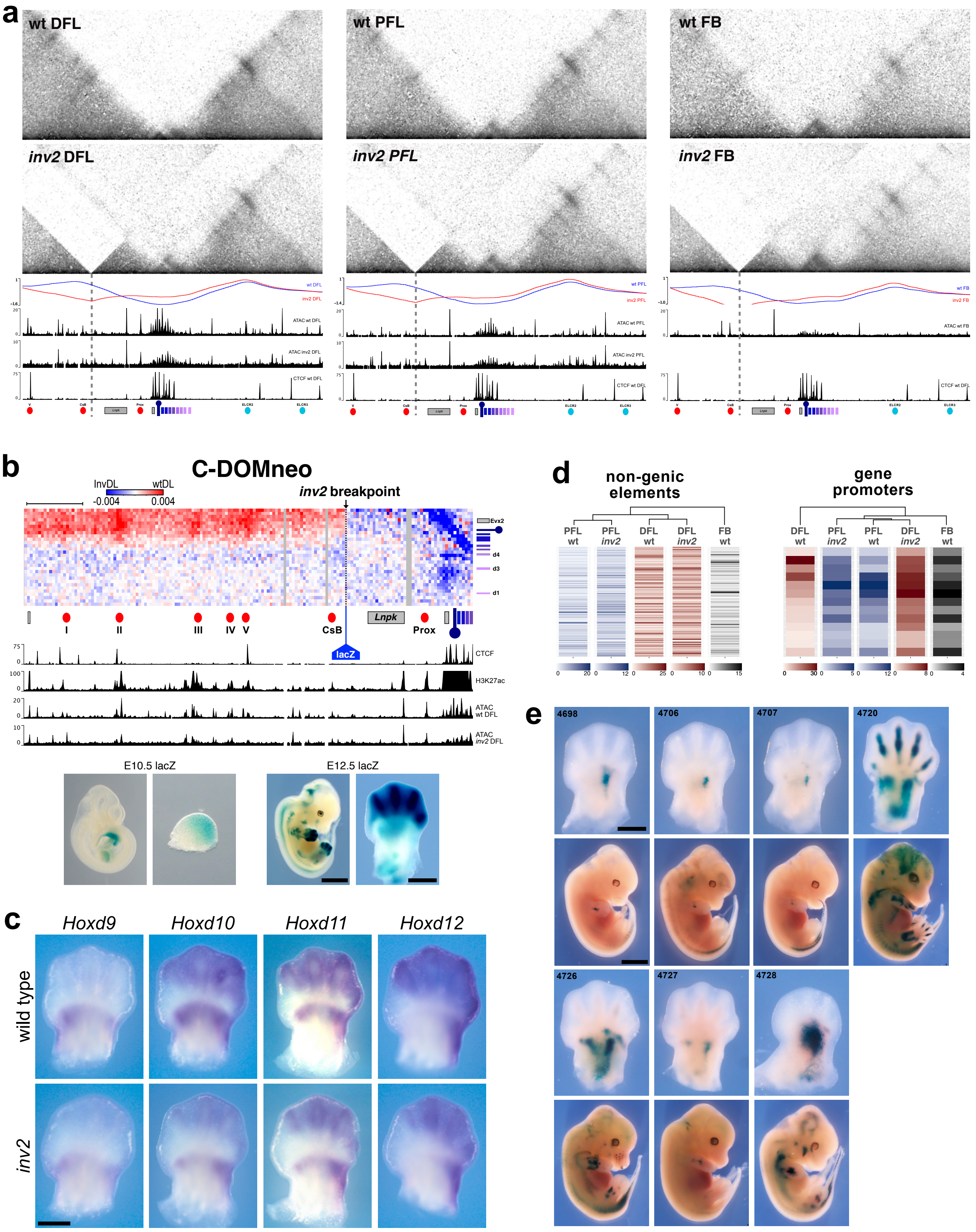

Bolt et al., 2021
Supplementary Figure 2

Supplementary Figure 2. Supplementary panels for experiments in Figure 2. a Control and mutant conditions for Capture Hi-C for the region around the *Hoxd* gene cluster (chr2:74277600-75147000). Bin size is 5kb, color scale is log transformed. CHi-C is mapped onto wild type mm10 in the *inv2* samples. The centromeric breakpoint position is indicated by a grey dashed line. The telomeric breakpoint is not visible at these coordinates. The red and blue lines below the CHi-C heatmaps are TAD-separation scores. The two ATAC-Seq tracks for the corresponding tissues (only DFL and PFL) are shown along with CTCF CUT&RUN for wt DFL. **b** Subtraction of contact frequencies in CHi-C in *inv2* distal limb (DL) samples from wild type DL. These contacts are mapped on the wild type mm10 genome comparing two regions (x-axis chr2:73900700-74703000 against y-axis chr2:74638000-74783000) demonstrates the changes in contacts between the *Hoxd* gene cluster and the C-DOM created by the *inv2* allele. Red indicates greater contact frequency in the wild type distal limb and blue is greater contact in the *inv2* distal limb. The inversion creates a novel regulatory environment that merges several proximal limb and distal limb enhancers. A *lacZ* sensor at the position of the inversion breakpoint responds to both types of enhancers and so the *lacZ* is detected in proximal and distal limb domains (bottom panel). **c** WISH on E12.5 forelimbs for *Hoxd* genes show very minor changes in gene expression in the limbs for these genes. *Hoxd12, Hoxd11,* and *Hoxd10* are reduced in the distal limb. In the proximal limb *Hoxd10* and *Hoxd11* are slightly reduced in the central region. On the posterior region of the proximal limb *Hoxd11* and *Hoxd12* show a very slight increase. Scale bar is 0.5mm. **d** Pearson correlation hierarchical clustering on the ATAC datasets in Figure 2. The color of each bin represents the average read density in that region. In the left panel, this analysis evaluates the ATAC profiles on the non-genic portions of the *HoxD* locus. Each sample clustered most closely based on tissue of origin. The right panel uses the same clustering analysis but only evaluating the ATAC profiles on accessible *Hoxd* gene promoters. The promoters of *Hoxd* genes in the two *inv2* samples cluster closely with the wild type PFL sample indicating changes in the accessibility of their promoters in the inversion configuration. **e** All replicate embryos collected from the PLE:lacZ transgenic experiment (Figure 2b). Scale bar for limbs is 0.5mm and for whole embryo is 2mm.

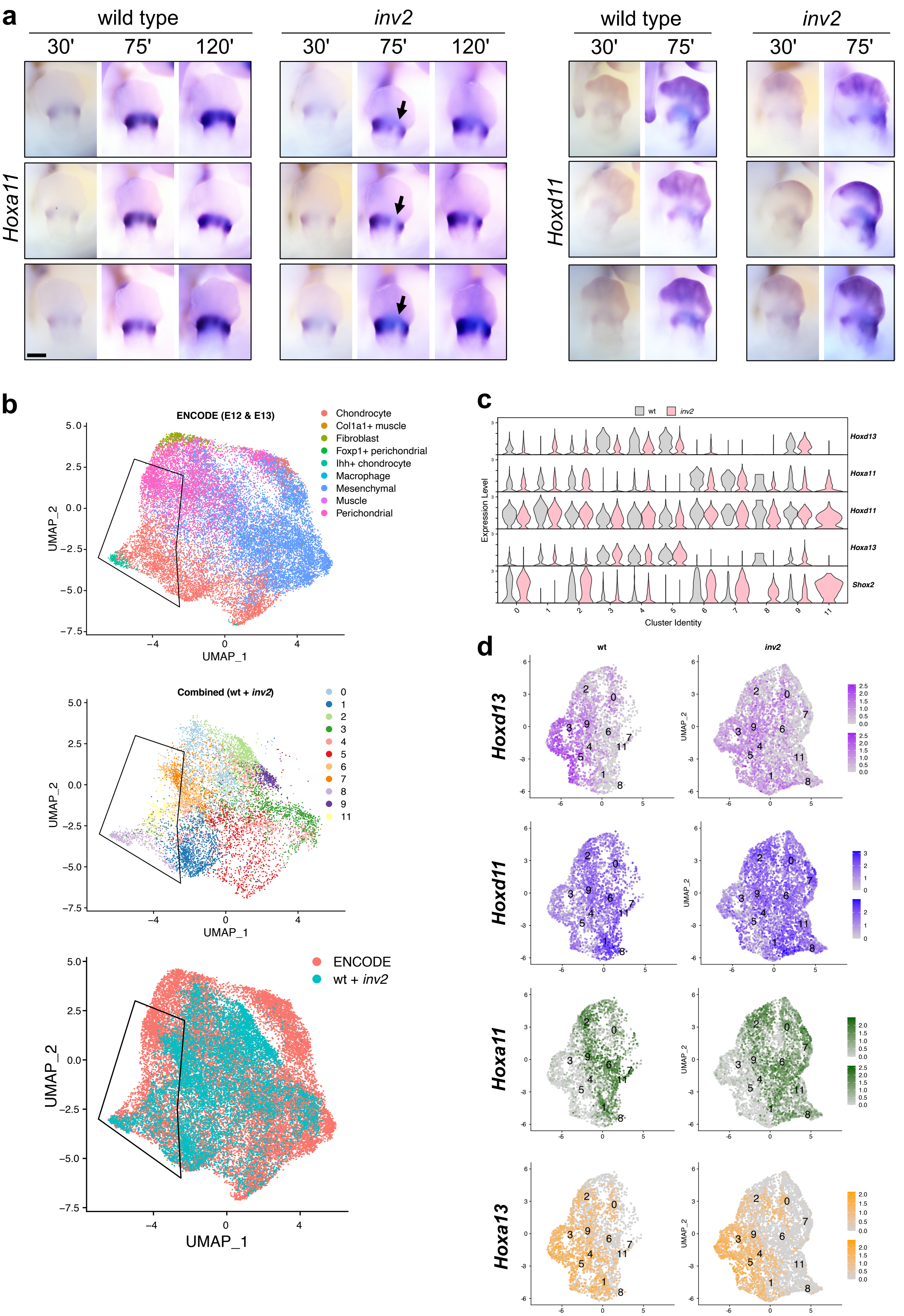

Bolt et al., 2021
Supplementary Figure 3

Supplementary Figure 3. Supplementary panels for experiments in Figure 3. a *In situ* hybridizations for *Hoxa11* and *Hoxd11* at E12.5 through a staining time-course. Each row of three images (*Hoxa11*) or two images (*Hoxd11*) is one embryo imaged three times. Wild type and *inv2* embryos were stained with *Hoxa11* or *Hoxd11* probes in triplicates. During the development of the stain, the embryos were photographed at 30 minutes, 75 minutes, and 120 minutes (*Hoxa11* only) in order to identify any region in the proximal limb that develops less or more slowly than the wild type limbs. In the *Hoxa11* samples a small region of the posterior portion of the limb bud stained very slowly (arrowhead). *Hoxd11* showed a weak increase in staining in the same position as the loss of *Hoxa11*, which is likely to arise due to a *cis*-effect from the inversion, not due to a trans-effect of the ectopic HOXD13 protein. Scale bar is 0.5mm. **b** Top: UMAP representation of wild type whole forelimbs at E12 and E13 scRNA-seq samples[1]. The black box encloses cell clusters corresponding to clusters 7, 8, and 11 in our data. Middle: our three scRNA-seq samples pooled together. Bottom: overlay of top and middle panels presented for comparison. The black box encloses clusters 7, 8, and 11 which are found in normal whole forelimbs. **c** Violin plots of the detected expression for the main clusters to determine which clusters have gained *Hoxd13* in the *inv2* samples but also express *Hoxa11* and *Hoxd11* in the wild type. *Hoxa13* was used to approximate distal limb clusters and *Shox2* for the proximal limb. **d** Expression of the genes used to determine the relevant proximal limb clusters displayed on UMAP.

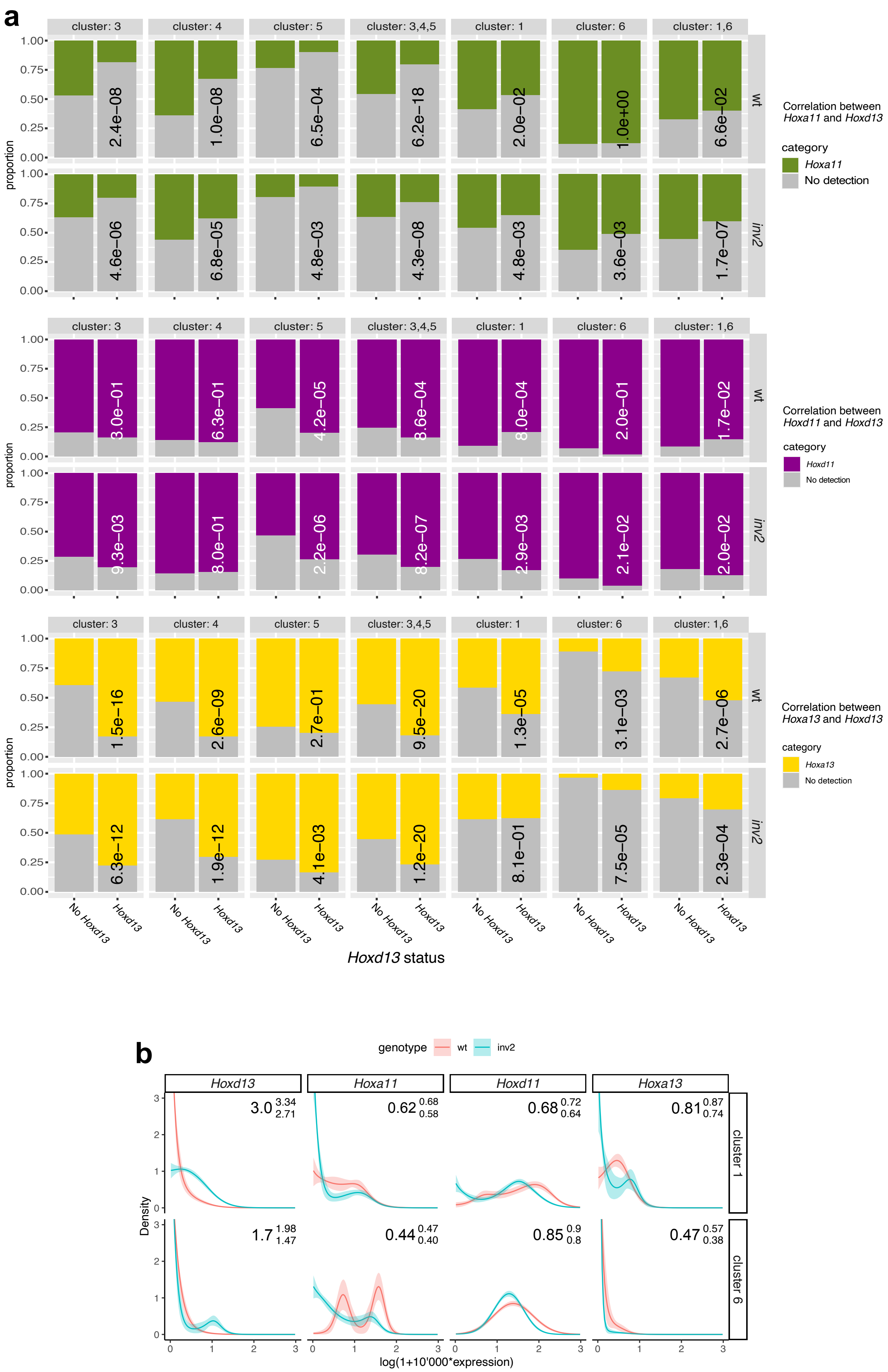

Bolt et al., 2021
Supplementary Figure 4

Supplementary Figure 4. Supplementary panels for additional analysis and baredSC. a Proportion of cells with detected UMI for *Hoxa11*, *Hoxd11*, and *Hoxa13* for each cluster and each genotype, in cells where *Hoxd13* is detected (*Hoxd13*) or not detected (No *Hoxd13*). Numbers indicate the p-value for the Fisher's exact test. **b** Distribution of inferred true expression of *Hoxd13*, *Hoxa11*, *Hoxd11* and *Hoxa13*, for cluster 1 and cluster 6 in each genotype. The solid line indicates the mean distribution while the shaded area indicates the 68% confidence interval. In the right corners are indicated an estimation and the 68% confidence interval of the fold-change of mean expression.

a

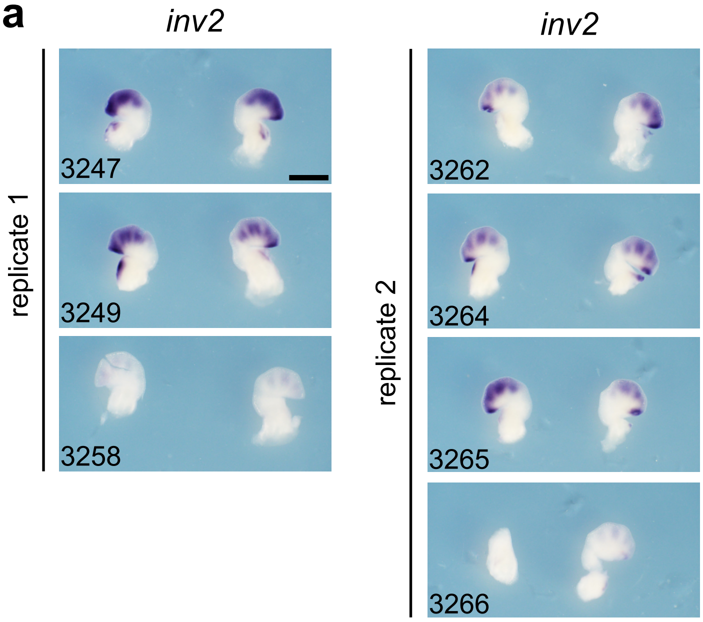

b

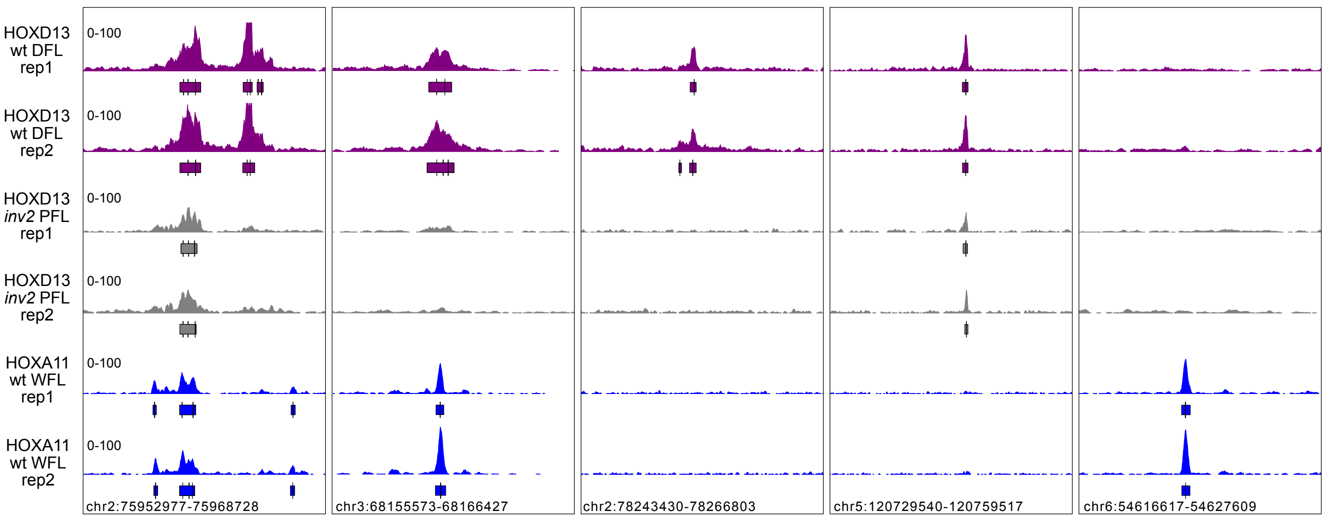

Bolt et al., 2021
Supplementary Figure 5

Supplementary Figure 5. Supplementary panels for the CUT&RUN experiment with HOXD13. a Limbs were kept after micro-dissection of the posterior proximal forelimb. They were then processed for WISH with a *Hoxd13* probe to estimate in retrospect how much distal limb contamination was present in the *inv2* replicates. The sample shaker failed during probe incubation leading to the variation in staining. Scale bar is 1mm. **b** Screenshots of genomic tracks for individual replicates of: (top, purple) HOXD13 in E12.5 wild type Distal Forelimbs, (middle, grey) HOXD13 in E12.5 *inv2* Posterior Proximal Forelimbs, and (bottom, blue) E11.5 whole forelimb HOXA11 ChIP-Seq datasets (GSM3504924 and GSM3504925)[2].

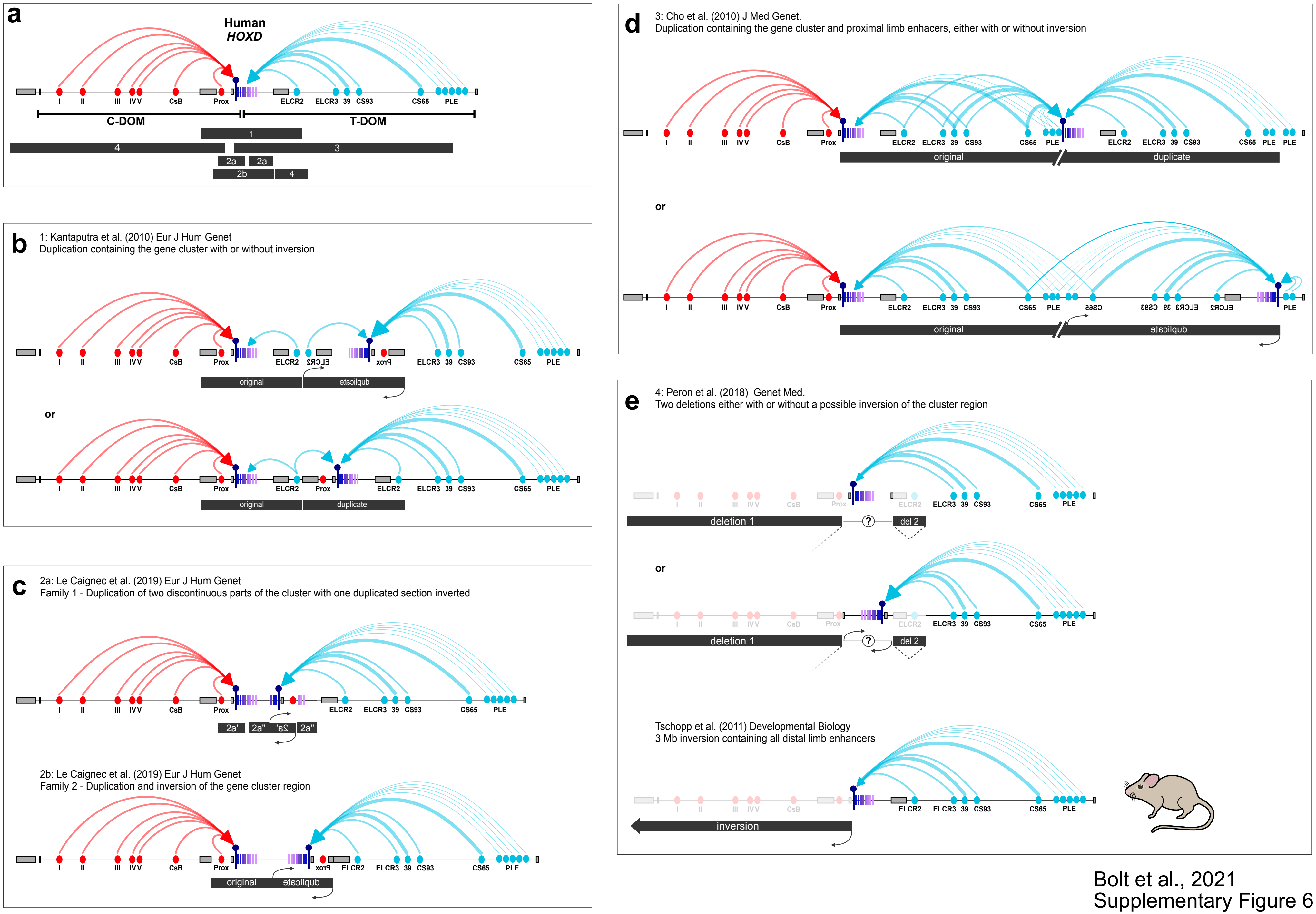

Bolt et al., 2021
Supplementary Figure 6

Supplementary Figure 6. An inclusive mechanistic model for all human mesomelic dysplasias associated with 2q31. a Recapitulated scheme of the human regulatory C-DOM and T-DOM landscapes flanking the *HOXD* cluster, with the distal (red) and proximal (blue) regulations. The black rectangles below indicate the positions of the various chromosomal rearrangement that are detailed under panels (**b**-**e**). **b** Kantaputra *et al.* (2010)[3] reported a duplication of unknown orientation (either top or bottom). In both cases, the duplicated copy of *HOXD13* (purple pin) is moved away of the distal enhancers, which continue to activate the native *HOXD13* copy, and positioned at the vicinity of the proximal enhancers, certainly leading to its ectopic expression in proximal limbs and concurrent mesomelic dysplasia. **c** In the two families reported by Le Caignec *et al.* (2019)[4], the same explanatory framework can be used. In the first family (top), a double duplication with one inversion brings *HOXD13* right next to the proximal limb enhancers, while distal enhancers are still involved in the regulation of the native *HOXD13* in distal cells. In family 2 (bottom), a simpler inverted duplication has the exact same effect. **d** The condition reported by Cho *et al.* (2010)[5] is slightly more complicated as a duplication of unknown orientation includes both the gene cluster and most of T-DOM i.e. the proximal regulatory landscape. In both cases, however, it is clear that *HOXD13* must fall under the control of proximal enhancers, either from both sides (top, non-inverted duplication), or from one side only (bottom, inverted duplication), leading again to a proximal gain of expression and concurrent mesomelic dysplasia. **e** The case reported by Peron *et al.* (2018)[6] involves two separate deletions, either in *cis* or in *trans* (undetermined). The larger deletion (deletion 1) removes all of the C-DOM containing the distal enhancers, whereas a shorter deletion (del 2) removes a piece of the T-DOM. Should the deletions be in *cis*, the orientation of the cluster located in between could be either way, as a result of the two deletions[7], thus leading to two potential configurations (top and middle). The first configuration (top) is very similar to an engineered allele produced in mice (bottom) whereby all distal enhancers were separated from the *HoxD* cluster through a large inversion[8]. Constitutive contacts between *Hoxd13* and some of these enhancers were released, thus allowing *Hoxd13* to interact with proximal enhancers and to be expressed in proximal cells leading to a weak mesomelic dysplasia [8,9]. The exact same phenomenon would likely occur in configuration 1 of Peron *et al.* (2018)[6]. Should the deletions be in *cis* and the cluster inverted (middle), the latter effect would be strengthened by an increased proximity between *HOXD13* and proximal enhancers.

Supplementary Table 1:
List of genotyping primers, CRISPR guide sequences, and enhancer elements.

Genotyping Primers

Allele	Forward	Reverse
wild type	296 (del(65)WT)<br>GCTGCCCTATGCACCAGT	297 (del(65)-1F)<br>TGACAGCTCGTTTTGTGCTC
HoxD^{inv2}	391 (301.3'.3)<br>CCCTGTCTTAGGTCAGTTAGGATCAC	500 (65.tel.1.rev)<br>GGGTTGACAGACCCAGTCTTATGC
wild type	655 (5'sgdel7/Afw1)<br>CCGGACATGTGCGACGGG	
Hoxd13^{hd}	656 (d13-3'rev1)<br>TGTCTGTGGCCAACCTGGCC	

CRISPR Guide Name	CRISPR Guide Sequence
Hoxd13_5'_gRNA10	CGGACATGTGCGTCTACCGA

Proximal Limb Enhancers (PLE) Coordinates (mm10)

CS68	chr2:75462086-75462878
PLE01	chr2:75470294-75470725
PLE02	chr2:75492186-75492863
PLE03	chr2:75496305-75496647
PLE04	chr2:75496731-75497336
PLE05	chr2:75557240-75557866

Constructed Sequence

```
>PLE_TgN
ggtaccAAGTATATCGAGGTAGATTGAAAACTCAAAGGACAAAGCTCTCATCTCAGGCAACTTTTCCTGCTACCTAACTCCACACAGCTATGACAG
CTGTGTGAGGAGAGCTTCTGTCCCCAGGTGACAGAAGCCACTGCTGGAGCTCTGGAACACTGCCCAGGCTTGCTCAGATCCACCCCAGGGCTTT
CCTTCCTCTGCCTCAGCTCCTCTCAGCCCCTCACTCATCCTTTTAAACCACACACTAGGGTCACTTGCTGAAATCCACCTGCCATGCCGCCATGCC
CTCTGCCTAGAATCACAGAGGCACTCTGAGCTTGTACACCTGCCCCATACCCTTAGACAGCTTACGAATTAGCTATCAGGAACTACGATCAATTT
TAGGAGCCCCCTGGGAGAAAGAACTCATTCAATATTGGCTGCATTTGTTTGACTCAACCTTAGGTAAGCTTTTCCATATGTCCAAGCTAAGGCCC
ATGCAAACTTACATTTGGCATCTTTCAAACAGATGCTCTTTAAATACTGGTTTTTATTTGTCCGGTGGAATCAATAAAACAgatgcatgcctgtaatcccagca
cttgggaggctgaggtaggaacatctcaagtttgagactatcttgggctatagaatgagactctgtagaaaagaataagaGGAGTGAAAATGGGGGTgggggtggaaaggtctccaggttgagaacatttgctgcccttcca
gaagacatgaattctgttcctgttgttctcagaacccagttcaggtggttgcaaactgcctgtatgccagctccgggCAGTTAGCCATTTTTCTTGAACAAGAACCTGGAAAATCTGTCATTCTA
TAGCTCACAAAGACACCCGTAATCTGCTATCAGCATACAGTTTGATAAAAGAGCCATTTCCCCTTTCTCACAATCTTCTACTGGCAGGAACAAG
AGCCCAGTGCGATCCAACGCCGAGCACATGGCATTTCTGGCTGTGATACAAAGGACTCCAACATCCTTCTGCATATTTGATGACCCTGGGGACC
CTGCAGTGTAGCGCTGCTGTGGGAACATCTGAGGCCGCCAGCGGGCACAGCTGTCTTTGTCTTCCCACGCTGCTCAGAGCTGTGGTTGTGGGGA
GCTGCAGAAACGGAAACGGGGTGCTGGTTGAGAAAAATCATAAGGCTCACCAGGCCCACATTCCCCCACGAAATCAGTCTATCTGGTTCTGAGT
TGCTGATacacaATGCCACCTAAATAAGCCTATTAAAAAGGACTCCAGTGGAGACACTGATTTGCCATAAACTGCAAAATGCTTCGGAGCTCAGCA
GAGACTGGCTCCTGTCACACACAGCCTGGCAGTGGCCCAGCTCCACACCAGTGCACACTTTTCCAATTACAAGCTGAAGATTCCTACGGGGACT
CAGGACTCTTGTTTCAGTCTTCCAGTGCAGGAGAAATTCTTCATGGAAGAAAGTGCCCATGGCTCAAGTTCTTCAAGCTCTGTAAAAGAGAGAC
CTACAGTTCCCACAACAGGGAAGATTCTCTTCTGTTTTCCTGGTTTTTTATTTTTTTCACATCTTAAATTCTGCAAATTCTTGAAGCAATGGAATG
AAAGTTGAATCCCCTAATGATGTGATAGGCACTCCTTATTTATTGACTGTTTGGAACAGCAAAGTCTAAACATTTGAGGCCAGGCGAAGTGTTC
ACATTCTGTTCTTTCCACAGCTTAGGGGTGAATACATAAAACCGCTGAAGGCAATTGAAGAACTTTAAAGGAAACCGGCCACAGGTGGGGGTG
CTCTTGATTAAAGACAGACAAAGTCATGTATTAAAAGTGGCTAGCTCAGGCTGAgtgcacctaccagtaatcgcagaactcaggggATAATATCTCACTTTGATTA
AAATAAGTGTCAAATATTCACCCGATAGTCCTATCCTTGTAGAGCGTATGTCTCAAACCACAAAGTGCTTATTCAtggttccagtatgaaacacctcatgcatgctcc
tgtgtttgagtgcttgcttcacaggtggagctgctgtttggaaattctgacgaacttttatgaggagatgacagactgggagaagtaggactctggggttgggcctcgtgttatagccttccctacttccagtcgaggctctctggacagccaata
tgatataagcaactgtcttatgtccccacctactgtgccttcccagccatgagagactggtaattaataCAGACTGTCCCTTCTGTGACCTCAAGCTGCTTGTAGGACTGTAAGGAGGCCTG
CATGCAAACCAGAAAGTCATTTCAGTGCCAAAAGCTGTTTATCTTGAGGGGCCAAGAATGATATTTCACAATGTGGGACTCCTTCGGGTCGTAC
ATTGCATCTGTAAGAATCATGGACCTAGTTATAGTTTCTTTTTAATTCAGGGCTGAGGCGGTATAATGAAAGCTTTCTTCTTGCTGGGCTCAATTT
CTTCTCAGGAAAAACAAACATGCCAAGGTGGCTGGCAACAAACCAAGTCAGAGCTGATTAGCATTAAACTTGTCCTGGAAGCAGGTAGCCCCA
CAGTGGGAACAGCACATGACTGTGGCCTGGCTCCCACAAGACCTCTGAGCATGGTCCTGGACCCTGGTAACGCCTTCTGAATCCAAAGCCAGTA
ATCTCTTCTACTGATGTAAAACCAAAAGCAATCTGACCATCGAGTCTGTGTGAGTCCATCACTAACCCTTCTGCCCAACTTGAGCTGTTCTGGAA
CATATAGCTTCAGAACCTTACCCATCTGCATCCTGCCATAAAGGCCCAGGAGCACCGGGGGCCTCCAAGCAGGCTTCTGCCTTGCCTTCCTCACC
AGCTAGGTCTGGGAGTAAGAGGAGTAGAGAGAGCCACTTTAGCACATTCTAGTTCAAATCACTAAAAGAAAATGTCTTTTGATGAGGTAGCCA
AGTTTCTGGCAATGAAGTGTGCTAGTTTTCAGGACCAGCTCCCTCCCTCTCCGAGGCATGGACTAAAATTCCTGAATAAAATATAAAAATACCA
CCGTAACTGCATCCACGTGAGACCTCAGTGGTAAGAAAGGCCCCTCTGACAGCAACAGCACACTGGCAGGACTCGGCGAACTTGAGCATGGTT
TGCACGCTCTGGAGACAGAAGACAAAGCACAGGGACCACCCGGGTTGGTGTGTCCCACATTACGACCTTCATAAAACTGAAGCCCACAGCACA
CCCTCCTCGAAGGCAGGAACTAACATAAACCCTCCTCTTCTCCCCTAGAAGCAACACGGAAAACCAACATCTCAAAACCTAGCAGTCAAGAGA
ACAGGCAGAACCAAGCCTGGGAATTTATGGCCAAAAGCTAAGCCTCGCACACATTTGCACACAAATTCATGTTTAAAGCTTCTTTAAAAAATAA
AAAATTAACCTGAAGGCAATGCTATGAGATTGGTCCTGGAGTAgggccc
```

Supplementary Table 1. The supplementary table contains: the genotyping primer sequences for the *HoxD*^{inv2} and *Hoxd13*^{hd} alleles, the CRISPR guide sequence used to generate the *Hoxd13*^{hd} allele, the genomic coordinates for the enhancer sequences used in the PLE:lacZ transgene assay, and the DNA sequence for the PLE:lacZ enhancer-reporter construct.

Supplementary Video 1: Micro-CT scans of adult left forearm skeletons. All skeletons are homozygous for the indicated genotype.

REFERENCES FOR SUPPLEMENTARY FILES

1. He, P. *et al.* The changing mouse embryo transcriptome at whole tissue and single-cell resolution. *Nature* **583**, 760–767 (2020).

2. Desanlis, I. *et al.* HOX13-dependent chromatin accessibility underlies the transition towards the digit development program. *Nat Commun* **11**, 2491 (2020).

3. Kantaputra, P. N. *et al.* Mesomelic dysplasia Kantaputra type is associated with duplications of the HOXD locus on chromosome 2q. *Eur J Hum Genet* **18**, 1310 (2010).

4. LeCaignec, C. *et al.* Fryns type mesomelic dysplasia of the upper limbs caused by inverted duplications of the HOXD gene cluster. *Eur J Hum Genet* 1–9 (2019) doi:10.1038/s41431-019-0522-2.

5. Cho, T.-J. *et al.* A dominant mesomelic dysplasia associated with a 1.0-Mb microduplication of HOXD gene cluster at 2q31.1. *J Med Genet* **47**, 638 (2010).

6. Peron, A. *et al.* Prenatal upper-limb mesomelia and 2q31.1 microdeletions affecting the regulatory genome. *Genet Med* **20**, 1 2 (2018).

7. Kragesteen, B. K., Duboule, D., Mundlos, S. & Spielmann, M. Response to Peron et al. *Genet Med* **20**, 1 2 (2018).

8. Tschopp, P. & Duboule, D. A regulatory ‘landscape effect’ over the HoxD cluster. *Dev Biol* **351**, 288 296 (2011).

9. Montavon, T., Thevenet, L. & Duboule, D. Impact of copy number variations (CNVs) on long-range gene regulation at the HoxD locus. *Proc National Acad Sci* **109**, 20204–20211 (2012).
