# Supplementary material for: Mesomelic dysplasias associated with the HOXD locus are caused by regulatory reallocations
Source: Nat Commun. 2021 Aug 18;12:5013. doi: 10.1038/s41467-021-25330-y (PMC8373931; doi:10.1038/s41467-021-25330-y)
Supplement: Supplementary file 3 — Description of Additional Supplementary Files [file 41467_2021_25330_MOESM3_ESM.docx]

Description of Additional Supplementary Files

Title: Supplementary Video 1

Description: Micro-CT scans of adult left forearm skeletons. All skeletons are homozygous for the indicated genotype.
