# Supplementary material for: Mesomelic dysplasias associated with the HOXD locus are caused by regulatory reallocations
Source: Nat Commun. 2021 Aug 18;12:5013. doi: 10.1038/s41467-021-25330-y (PMC8373931; doi:10.1038/s41467-021-25330-y)
Supplement: Supplementary file 5 — Reporting Summary [file 41467_2021_25330_MOESM5_ESM.pdf]

nature research

Corresponding author(s): Christopher Chase Bolt
Denis Duboule

Last updated by author(s): 2021.07.21

Reporting Summary

Nature Research wishes to improve the reproducibility of the work that we publish. This form provides structure for consistency and transparency in reporting. For further information on Nature Research policies, see our Editorial Policies and the Editorial Policy Checklist.

Statistics

For all statistical analyses, confirm that the following items are present in the figure legend, table legend, main text, or Methods section.

n/a	Confirmed	
☐	☒	The exact sample size (*n*) for each experimental group/condition, given as a discrete number and unit of measurement
☐	☒	A statement on whether measurements were taken from distinct samples or whether the same sample was measured repeatedly
☐	☒	The statistical test(s) used AND whether they are one- or two-sided<br>*Only common tests should be described solely by name; describe more complex techniques in the Methods section.*
☒	☐	A description of all covariates tested
☐	☒	A description of any assumptions or corrections, such as tests of normality and adjustment for multiple comparisons
☐	☒	A full description of the statistical parameters including central tendency (e.g. means) or other basic estimates (e.g. regression coefficient) AND variation (e.g. standard deviation) or associated estimates of uncertainty (e.g. confidence intervals)
☐	☒	For null hypothesis testing, the test statistic (e.g. *F*, *t*, *r*) with confidence intervals, effect sizes, degrees of freedom and *P* value noted<br>*Give P values as exact values whenever suitable.*
☒	☐	For Bayesian analysis, information on the choice of priors and Markov chain Monte Carlo settings
☒	☐	For hierarchical and complex designs, identification of the appropriate level for tests and full reporting of outcomes
☐	☒	Estimates of effect sizes (e.g. Cohen's *d*, Pearson's *r*), indicating how they were calculated

Our web collection on statistics for biologists contains articles on many of the points above.

Software and code

Policy information about availability of computer code

Data collection

Olympus cellSens Standard 2.1 was used for image capture of embryos and skeletons.

Data analysis

Horos 3.3.6 was used for analysis of the microCT scans and bone measurements.
DataGraph 4.6.1 was used to generate box plots in figure 1c and calculate p-values.

For ATAC-seq analysis:
Bowtie2 version 2.3.4.1 was used to map the reads
bamtools version 2.4.1 was used to remove poor mapping quality reads.
Picards version 1.56.0 was used to remove duplicates.
Bedtools 2.18.2 was used to convert bam to bed.
A custom python script available at https://github.com/lldelisle/scriptsForBoltEtAl2021 was used to get the coverage.
MACS2 version 2.1.1.20160309 was used to call peaks.
Bedtools 2.27.1 was used to intersect the ATAC-seq peaks with different annotations
R 3.6.0 was used to plot the clustering.

For capture Hi-C analysis:
HiCUP version 0.6.1 was used to process the fastq with Bowtie2 version 2.2.6 and samtools version 1.2.
A custom python script available at https://github.com/lldelisle/scriptsForBoltEtAl2021 was used to convert the bam file to a valid pair file.
Cooler version 0.7.4 was used to generate matrices as cool file.
A custom python script available at https://github.com/lldelisle/scriptsForBoltEtAl2021 was used to convert the cool file to a text file.
R version 3.6.3 was used to plot the differential heatmaps.
HiCExplorer 3.5.1 was used to determine the level of insulation at the HoxD boundary in S. Figure 2a.

For CUT&RUN/ChIP analysis:
seqtk version 1.3.9 was used to subsample the ChIP
cutadapt version 1.16 was used to remove adapters
Bowtie2 2.3.4.1 was used for mapping
Samtools 1.2 was used for filtering.
Picards version 1.56.0 was used to remove duplicates.
Bedtools 2.18.2 was used to convert the bam file to bed.
MACS2 version 2.1.1.20160309 was used for peak calling.
HOMER 4.10 was used for motif finding.
Bedtools 2.27.1 was used to identify HOXD13 and HOXA11 binding site overlaps.
Diffbind 2.14.0 was used for differential binding analysis of HOXD13 and HOXA11 with DESeq2 1.24.0 and R version 3.6.

For single-cell RNAseq analysis:
Cell Ranger 3.1.0 was used for scRNA-seq mapping and counting.
Seurat 3.2.3 was used for analysis of the single cell RNA-seq with R 3.6.3.
Seurat 4.0.1 was used for the integration with R 4.0.
baredSC version 1.0.0 was used for correlation and fold-change estimation.
pygenometracks 3.3 was used to generate genomic tracks figures.
deepTools 3.5 was used for hierarchical cluster, and ATAC and CUT&RUN binding heatmaps.

For manuscripts utilizing custom algorithms or software that are central to the research but not yet described in published literature, software must be made available to editors and reviewers. We strongly encourage code deposition in a community repository (e.g. GitHub). See the Nature Research guidelines for submitting code & software for further information.

Data

Policy information about availability of data

All manuscripts must include a data availability statement. This statement should provide the following information, where applicable:

- Accession codes, unique identifiers, or web links for publicly available datasets
- A list of figures that have associated raw data
- A description of any restrictions on data availability

The data generated in this study are available as raw and processed datasets in the Gene Expression Omnibus (GEO) repository under accession number GSE165495 [https://www.ncbi.nlm.nih.gov/geo/query/acc.cgi?acc=GSE165495]. The mouse E11.5 HOXA11 ChIP-seq dataset was obtained from the GEO under SRR8290670 of GSM3504924 [https://www.ncbi.nlm.nih.gov/sra?term=SRX5105273] and SRR8290672 of GSM3504925 [https://www.ncbi.nlm.nih.gov/sra?term=SRX5105274]. These data are associated with Figs 2, 3, 4 and Supplementary Figs 2, 3, 4, and 5.

Field-specific reporting

Please select the one below that is the best fit for your research. If you are not sure, read the appropriate sections before making your selection.

☒ Life sciences ☐ Behavioural & social sciences ☐ Ecological, evolutionary & environmental sciences

For a reference copy of the document with all sections, see nature.com/documents/nr-reporting-summary-flat.pdf

Life sciences study design

All studies must disclose on these points even when the disclosure is negative.

Sample size

For all WISH, at least 3 biological replicates were evaluated (3 embryos), which is a commonly accepted sample size for such experiments. A sample size of 3 can demonstrate that a change in gene expression is consistent across genotypes, while balancing for animal usage considerations. For the figures, one embryo was chosen as representative of the set. For skeleton preparations, 3 biological replicates were used for wild type and inv2 mutants, measuring both forearms of each animal. For the 13hd and inv2:13hd, two biological replicates were used, measuring both forearms of each animal. For replicates in CUT&RUN, see the ChIP-seq section details below. For CHiC-seq and CTCF CUT&RUN-seq, the tissue was pooled from 6 or 7 embryos (both pairs of limbs) of the same genotype in order to produce enough material for experimentation. As these experiments result from pools of cells from several embryos, the resulting data is an average of measurements across many samples. In the scRNA-seq, one wild type embryo was used, with the PPFL collected from both forearms, and for each inv2 sample, the same dissection was performed, in two distinct embryos. Due to the cost of scRNA-seq experiments, we reasoned that a single replicate for wild type, and two replicates for the inv2 were sufficient to identify transcriptional changes that were significant. For the PLE:lacZ enhancer reporter transgenic embryos, we performed two rounds of injection into embryos. We stopped when we observed that 7 out of 7 embryos produced very similar staining in the proximal forelimb.

Data exclusions

No data was excluded from this study.

Replication

All experiments were performed in biological replicate when the results were likely to have a meaningful impact on the interpretation of the result. Skeletal preparations were performed on at least three biological replicates per genotype at each stage (Replication was successful). For the lacZ transgenic embryos carrying the PLE enhancers, we collected 34 viable embryos and 7 of them stained for lacZ; all of these embryos were included in Supplementary Figure 2e (Replication was successful). In the single cell RNA-seq experiment, one wild type sample was used and two biological replicates (coming from two different embryos) were used for the Inv2 sample (Replication was successful). The HOXD13 CUT&RUN experiment was produced in biological replicate to identify binding positions that are common across more than one pool of embryos (Replication was successful). ATAC-Seq and Capture Hi-C experiments were performed on pools of tissue, but performed only as

singletons. All WISH samples were performed in >3 replicates for each probe.

Randomization	For skeleton preparations (Blue/Red and microCT), the analysis was performed by blinding the person doing the measurements from sample identifications and genotypes until after measurements were made. For all other experiments, the genotypes of individual embryos were determined prior to experiments. Embryos were grouped by genotype for processing in experiments.
Blinding	For the measurement of bone lengths in Figure 1, the person doing the measurements was blinded to the genotype of all animals until all measurements were completed. For all other experiments, it was necessary to know the genotype of individual embryos before processing through experimental protocols. So in this case, the embryos were genotyped and then dissected (and pooled if necessary) for experiments.

Reporting for specific materials, systems and methods

We require information from authors about some types of materials, experimental systems and methods used in many studies. Here, indicate whether each material, system or method listed is relevant to your study. If you are not sure if a list item applies to your research, read the appropriate section before selecting a response.

Materials & experimental systems

n/a	Involved in the study	
☐	☒	Antibodies
☒	☐	Eukaryotic cell lines
☒	☐	Palaeontology and archaeology
☐	☒	Animals and other organisms
☒	☐	Human research participants
☒	☐	Clinical data
☒	☐	Dual use research of concern

Methods

n/a	Involved in the study	
☐	☒	ChIP-seq
☒	☐	Flow cytometry
☒	☐	MRI-based neuroimaging

Antibodies

Antibodies used	anti-HOXD13 Abcam Ab19866 Lot#GR3290844-1, anti-CTCF Active Motif 61311 Lot#17118005
Validation	anti-HOXD13 antibody was validated in mouse E12.5 distal forelimb cells from embryos homozygous for a frame shift mutation (reported in this manuscript) in the Hoxd13 gene that ablates DNA binding. These cells were used for HOXD13 CUT&RUN and validated for no enrichment by qPCR.<br>The CTCF antibody has been extensively tested and validated in mouse tissues with ChIP-qPCR, ChIP-seq, immunohistochemistry, and Western blot. See manufacturers website (https://www.activemotif.com/catalog/details/61311/ctcf-antibody-pab).

Animals and other organisms

Policy information about studies involving animals; ARRIVE guidelines recommended for reporting animal research

Laboratory animals	Mus musculus, all animals are kept in a continous back cross with BL6 X CBA F1 hybrids. Sex of the animals was not considered in this analysis. All embryos used for WISH were at E12.5 unless otherwise explicitly indicated in the figures (Fig 1d). CHi-C-seq, CUT&RUN-seq, ATAC-seq, and scRNA-seq were performed on E12.5 embryos. Mice were housed in the University of Geneva Sciences III animalarie with light 07:00-19:00 in the summer, and 06:00-18:00 in winter with ambient temperatures maintained between 22-23ºC and 45-55% humidity, the air is renewed 17-times per hour.
Wild animals	No wild animals were used in this study.
Field-collected samples	No field-collected samples were used in this study.
Ethics oversight	All experiments were performed in agreement with the Swiss Law on Animal Protection (LPA) under license numbers GE 81/14 and VD2306.2 (to D. Duboule).

Note that full information on the approval of the study protocol must also be provided in the manuscript.

ChIP-seq

Data deposition

☒ Confirm that both raw and final processed data have been deposited in a public database such as GEO.

☒ Confirm that you have deposited or provided access to graph files (e.g. BED files) for the called peaks.

Data access links<br>*May remain private before publication.*	The data generated in this study are available as raw and processed datasets in the Gene Expression Omnibus (GEO) repository under accession number GSE165495 [https://www.ncbi.nlm.nih.gov/geo/query/acc.cgi?acc=GSE165495].<br>All scripts necessary to reproduce figures from raw data (including custom scripts) are available at GitHub [https://doi.org/10.5281/zenodo.5118344].

Files in database submission

./CUTandRUN/E12.5_inv2_DFL_CR_CTCF.narrowPeak
./CUTandRUN/E12.5_wt_DFL_CR_CTCF_R1.fastq.gz
./CUTandRUN/E12.5_inv2_PPFL_CR_HOXD13_rep1.narrowPeak
./CUTandRUN/E12.5_inv2_PPFL_CR_HOXD13_rep2_R1.fastq.gz
./CUTandRUN/E12.5_inv2_PPFL_CR_HOXD13_rep1_R2.fastq.gz
./CUTandRUN/E12.5_wt_PFL_CR_CTCF.bw
./CUTandRUN/E12.5_wt_PFL_CR_CTCF_R1.fastq.gz
./CUTandRUN/E12.5_wt_FB_CR_CTCF.bw
./CUTandRUN/E12.5_inv2_PFL_CR_CTCF_R1.fastq.gz
./CUTandRUN/E12.5_wt_DFL_CR_HOXD13_rep2_R1.fastq.gz
./CUTandRUN/E12.5_wt_DFL_CR_HOXD13_rep1_R2.fastq.gz
./CUTandRUN/E12.5_inv2_DFL_CR_CTCF.bw
./CUTandRUN/E12.5_inv2_DFL_CR_CTCF_R1.fastq.gz
./CUTandRUN/E12.5_wt_DFL_CR_CTCF.narrowPeak
./CUTandRUN/E12.5_wt_FB_CR_CTCF.narrowPeak
./CUTandRUN/E12.5_wt_FB_CR_CTCF_R1.fastq.gz
./CUTandRUN/E12.5_wt_DFL_CR_HOXD13_rep1.narrowPeak
./CUTandRUN/E12.5_wt_FB_CR_CTCF_R2.fastq.gz
./CUTandRUN/E12.5_inv2_PFL_CR_CTCF.narrowPeak
./CUTandRUN/E12.5_inv2_DFL_CR_CTCF_R2.fastq.gz
./CUTandRUN/E12.5_wt_DFL_CR_HOXD13_rep1_R1.fastq.gz
./CUTandRUN/E12.5_wt_DFL_CR_HOXD13_rep2_R2.fastq.gz
./CUTandRUN/E12.5_inv2_PFL_CR_CTCF.bw
./CUTandRUN/E12.5_inv2_PPFL_CR_HOXD13_rep2.narrowPeak
./CUTandRUN/E12.5_inv2_PFL_CR_CTCF_R2.fastq.gz
./CUTandRUN/E12.5_wt_PFL_CR_CTCF_R2.fastq.gz
./CUTandRUN/E12.5_inv2_PPFL_CR_HOXD13_rep1_R1.fastq.gz
./CUTandRUN/E12.5_inv2_PPFL_CR_HOXD13_rep2_R2.fastq.gz
./CUTandRUN/E12.5_wt_DFL_CR_CTCF.bw
./CUTandRUN/E12.5_inv2_PPFL_CR_HOXD13_rep1.bw
./CUTandRUN/E12.5_wt_DFL_CR_HOXD13_rep2.narrowPeak
./CUTandRUN/E12.5_wt_DFL_CR_HOXD13_rep2.bw
./CUTandRUN/E12.5_wt_DFL_CR_CTCF_R2.fastq.gz
./CUTandRUN/E12.5_wt_PFL_CR_CTCF.narrowPeak
./CUTandRUN/E12.5_wt_DFL_CR_HOXD13_rep1.bw
./CUTandRUN/E12.5_inv2_PPFL_CR_HOXD13_rep2.bw

Genome browser session
(e.g. UCSC)

No longer applicable

Methodology

Replicates

CTCF CUT&RUN was not performed in replicate. HOXD13 CUT&RUN was performed in biological replicates. In wild type distal forelimb replicate 1, 4 pairs of distal forelimbs were used. In replicate 2, 4 pairs of distal forelimbs were used. For the inv2 PPFL samples, the posterior proximal forelimb was dissected. For replicate 1, the PPFL was collected from both forelimbs from three inv2 mutants. For replicate 2, the PPFL was collected from both forelimbs of 4 inv2 embryos.

Sequencing depth

CTCF wt PFL: sequenced to 28 mio paired-end reads at 35 bp per end
CTCF wt DFL: sequenced to 25 mio paired-end reads at 35 bp per end
CTCF wt FB: sequenced to 16 mio paired-end reads at 35 bp per end
CTCF inv2 PFL: sequenced to 30 mio paired-end reads at 35 bp per end
CTCF inv2 DFL: sequenced to 33 mio paired-end reads at 35 bp per end
HOXD13 wt DFL rep1: sequenced to 25 mio paired-end reads at 35 bp per end
HOXD13 wt DFL rep2: sequenced to 25 mio paired-end reads at 35 bp per end
HOXD13 inv2 PPFL rep1: sequenced to 26 mio paired-end reads at 35 bp per end
HOXD13 inv2 PPFL rep2: sequenced to 25 mio paired-end reads at 35 bp per end

Antibodies

anti-HOXD13 Abcam Ab19866 Lot#GR3290844-1, anti-CTCF Active Motif 61311 Lot#17118005

Peak calling parameters

```
# bowtie2  version 2.3.4.1
bowtie2  -p ${GALAXY_SLOTS:-4}  -x 'mm10_UCSC'   -1 'input_f.fastq.gz' -2 'input_r.fastq.gz' -I 0 -X 1000 --fr --no-mixed --no-discordant --dovetail   --very-sensitive  2> 'mapping_stat'  | samtools sort -@${GALAXY_SLOTS:-2} -O bam -o 'output.bam'

# samtools version 1.2
samtools view -o 'filtered.bam' -h   -b  -q 30 -f 0x2 input.bam 2>&1

# Picards version 1.56.0:
python devteam/picard/bf1c3f9f8282/picard/picard_wrapper.py -i "filtered.bam" -n "Dupes Marked " --tmpdir "/tmp" -o "rmdup.bam" --remdups "true" --assumesorted "true" --readregex "[a-zA-Z0-9]+:[0-9]:([0-9]+):([0-9]+):([0-9]+).*" --optdupdist "100" -j "$JAVA_JAR_PATH/MarkDuplicates.jar" -d "output" -t "output.txt" -e "bam"
```

```
# Bedtools version 2.18,2
bedtools bamtobed  -i ./input.bam > output.bed

# macs2 version 2.1.1.20160309
macs2 callpeak  --name 'MACS2'   -t 'output.bed'    --format BED   --gsize '1870000000'        --call-summits  --keep-dup '1'   --bdg  --qvalue '0.05'  --nomodel --extsize '200' --shift '-100'  2>&1 > macs2_stderr
```

Data quality

For CTCF CUT&RUN, between 40 000 and 50 000 peaks were found with FDR less than 5% and fold-change above 5. For HOXD13 CUT&RUN, it was around 1200 in the inv2 samples and 4000-5000 in the wt samples.

Software

baredSC for scRNA analysis. Lopez-Delisle, L. & Delisle, J.-B. baredSC: Bayesian Approach to Retrieve Expression Distribution of Single-Cell. bioRxiv 2021.05.26.445740 (2021) doi:10.1101/2021.05.26.445740.
